# How Parkinsonian Toxins Dysregulate the Autophagy Machinery

**DOI:** 10.3390/ijms141122163

**Published:** 2013-11-08

**Authors:** Ruben K. Dagda, Tania Das Banerjee, Elzbieta Janda

**Affiliations:** 1 Department of Pharmacology, University of Nevada School of Medicine, Manville Building 18A, Reno, NV 89557, USA; E-Mail: tdasbanerjee@medicine.nevada.edu; 2 Department of Health Sciences, Magna Graecia University, Campus Germaneto, 88100 Cantazaro, Italy; E-Mail: janda@unicz.it

**Keywords:** MPTP, 6-hydroxydopamine, rotenone, paraquat, methamphetamine, autophagy, mitophagy, Parkinson’s disease

## Abstract

Since their discovery, Parkinsonian toxins (6-hydroxydopamine, MPP+, paraquat, and rotenone) have been widely employed as *in vivo* and *in vitro* chemical models of Parkinson’s disease (PD). Alterations in mitochondrial homeostasis, protein quality control pathways, and more recently, autophagy/mitophagy have been implicated in neurotoxin models of PD. Here, we highlight the molecular mechanisms by which different PD toxins dysregulate autophagy/mitophagy and how alterations of these pathways play beneficial or detrimental roles in dopamine neurons. The convergent and divergent effects of PD toxins on mitochondrial function and autophagy/mitophagy are also discussed in this review. Furthermore, we propose new diagnostic tools and discuss how pharmacological modulators of autophagy/mitophagy can be developed as disease-modifying treatments for PD. Finally, we discuss the critical need to identify endogenous and synthetic forms of PD toxins and develop efficient health preventive programs to mitigate the risk of developing PD.

## 1. Introduction

Autophagy is the coordinated catabolic process by which damaged and aged organelles are directed for lysosomal-mediated degradation by acid hydrolases, reviewed in [1,2]. To this date, three types of autophagy have been identified: macroautophagy, microautophagy, and chaperone-mediated autophagy. Although macroautophagy was once thought to be a promiscuous process, several studies have identified autophagy pathways that specifically target certain organelles including peroxisomes (pexophagy), ER (ERphagy), and mitochondria (termed mitophagy), reviewed in [1,3–5].

The autophagy machinery is an ancient and well conserved cellular system for regulating the turnover of damaged organelles that was initially discovered in yeast. Macroautophagy is triggered by starvation (glucose deprivation or serum withdrawal) or stimulated pharmacologically by rapamycin, an inhibitor of target of rapamycin (mTOR) pathway. Over 36 different autophagy-related genes (ATG) and 41 other non-ATG proteins initially identified in yeast are known to regulate autophagy. While macroautophagy can non-selectively degrade large protein aggregates and cytosolic components, mitophagy is a highly specific process in mammalian cells. Indeed, a plethora of scientific evidence over the past 10 years has shown that mitophagy is a specific process for selectively removing damaged and aged mitochondria that is coordinated by a variety of cytosolic and mitochondrial intermediate players and downstream “effectors” responsible for selectively recognizing the damaged cargo [6–9].

Autophagy was once believed to be dispensable in the central nervous system given that neurons are refractory to glucose starvation *in vivo*. However, this view was challenged seven years ago when two landmark studies demonstrated that gene deletions of ATG5 and ATG7 produce widespread brain degeneration in mice suggesting that autophagy plays a prosurvival role in the central nervous system [10,11]. On the other hand, chronic oxidative stress induced by many neurodegenerative diseases including amyotrophic lateral sclerosis (ALS), Alzheimer’s disease (AD), Huntington’s disease (HD), prion-related diseases (PRD), and Parkinson’s disease (PD) lead to early dysregulation of autophagy [12–14].

Autophagy can be a double-edged sword with both beneficial and detrimental roles in neurons. On the other hand, either a prolonged increase or decrease in autophagic flux equally contributes to the progression of neurodegenerative diseases. For instance, aberrant increases in early autophagic vacuole (AV) numbers and decreased Beclin-1 (a positive modulator of autophagy) levels have been observed in neurons from AD postmortem brain tissue suggesting that a disruption of autophagic flux contributes to an accumulation of fibrillar protein aggregates and degeneration of affected neurons [14]. Conversely, an increase in the number of mature AVs is observed in the neurites and somas of neurons of PD postmortem brain tissue suggesting that an aberrant upregulation of autophagy contributes to PD pathogenesis [15]. A caveat to studying autophagy in human brain tissue by ultrastructural analyses is the difficulty for determining whether an accumulation of AVs is a consequence of an aberrant upregulation of autophagy or a decreased autophagic flux in human brain tissue. Cell culture and rodent chemical models of PD have shown that treating animals with MPTP or neuronal cells with MPP+—the active metabolite of MPTP—leads to enhanced autophagy [16,17]. MPP+ also induces mitophagy, a pathological event that is detrimental to survival as it depletes neurons of critical mitochondrial ATP stores and blocks mitochondrial biogenesis [17,18]. Conversely, treating neurons with the pesticide paraquat impairs autophagic flux leading to an aberrant accumulation of dysfunctional mitochondria and induction of α-synucleopathies [19,20]. Hence, either overactive autophagic flux in the absence of biogenesis or impaired autophagy produces a similar detrimental effect on neuronal survival, reviewed in [21].

### Overview on the Selective Sensitivity of Dopamine Neurons to Oxidative Stress

PD is a relentless, chronic, incurable movement disorder that affects approximately 1% of the North American population, reviewed in [22]. PD is characterized by the progressive loss of dopamine neurons that project from the midbrain to the caudate and putamen of the striatum, leading to de-repression of inhibitory GABAergic pathways that project from the thalamus to the cortex and a loss of motor function [23]. A pathological hallmark of PD includes the presence of large protein aggregates termed Lewy bodies which are predominantly composed of α-synuclein, ubiquitin, Parkin, PTEN-induced kinase-1 (PINK1), and other less abundant proteins [22,24,25]. Although the majority of PD cases are caused by environmental factors or unknown factors (termed idiopathic or sporadic cases), approximately 10% of PD cases are caused by mutations in over 12 different genes involved in the regulation of proteasomal degradation pathways (Parkin, UCHL-1), mitochondrial homeostasis (PINK1, Omi/Htra, DJ-1, LRRK2), antioxidant response pathways (DJ1), lysosome function (ATP13A2), and mitophagy (PINK1 and Parkin) [25,26]. While PD remains an incurable disease, the tremors and bradykinesia (impaired ability to initiate movement) can be successfully but temporarily reversed by treating patients with l-dopamine (l-DOPA) in combination with carbidopa or with a combination of different dopamine agonists and monoamine oxidase-B (MOA-B) inhibitors to treat severe bouts of dyskinesias. However, by the time a patient is diagnosed with PD symptoms, more than 90% of midbrain neurons have degenerated which underscores the need to develop new diagnostic tools or biomarkers for diagnosing PD in its early stages [23].

**Figure 1:**
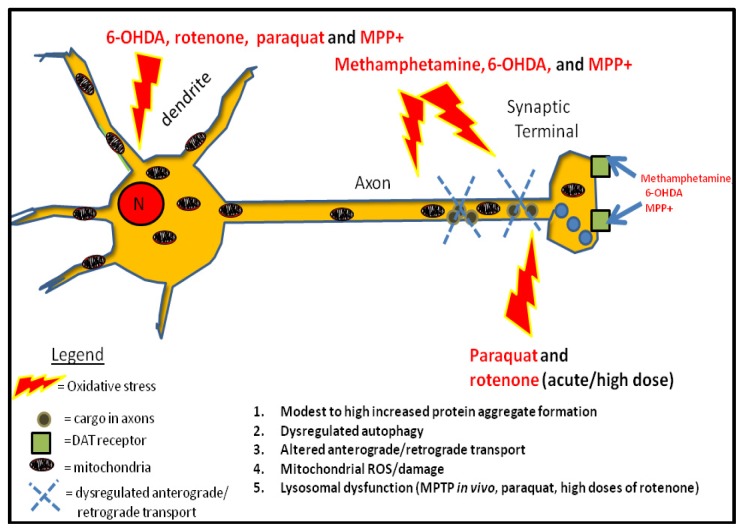
Compiled figure on the effects of Parkinson’s disease (PD) toxins on neuronal homeostasis. Parkinsonian toxins converge on their ability to damage mitochondria, alter bidirectional organellar transport across axons but differ on the molecular mechanism(s) by which they dysregulate autophagy (please refer to Section 3 of review). Toxins such as 6-hydroxydopamine (6-OHDA), methamphetamine, MPP+ and low doses of rotenone can increase ROS levels, damage mitochondria, elevate AV numbers and cause modest accumulation of protein aggregates including α-synuclein. Conversely, paraquat and high doses of rotenone and MPTP *in vivo* can promote mitochondrial dysfunction, compromise lysosomal integrity, increase protein aggregation due to impaired basal autophagy, and promote the loss of neurites and soma. 6-OHDA, methamphetamine, and MPP+ interact with DAT receptors (blue arrows) for uptake (6-OHDA and MPP+) or to induce release of dopamine (methamphetamine). Unlike other PD toxins, methamphetamine selectively degenerates neurites while sparing the cell bodies. All toxins can alter retrograde/anterograde transport (indicated by dashed X’s), and recapitulate PD pathogenesis *in vivo* including motor dysfunction and *substantia nigra* degeneration.

Midbrain neurons are particularly sensitive to oxidative stress induced by PD toxins compared to other neuronal populations or cell types. Some studies have suggested that the high neuromelanin content, high dopamine levels, and low mitochondrial content render midbrain neurons highly vulnerable to oxidative stress due to their high dopamine metabolism [27]. While a majority of PD toxins can cause a rapid loss of synaptic connectivity and cell death *in vitro*, the slow and progressive nature of degeneration of midbrain neurons in PD may depend on their ability to upregulate compensatory autophagy pathways to cope with the high oxidative stress contributed by dysfunctional mitochondria, reviewed in [12,28].The following sections of this review will provide a broad overview of the specific mechanisms by which PD toxins dysregulate autophagy/mitophagy and the neuronal survival pathways that are interrupted by chronic oxidative stress induced by toxins (Figure 1).

## 2. Mechanisms of Dysregulated Autophagy and Mitophagy by Parkinsonian Toxins

Autophagy is a catabolic cellular process that is initiated by a coordinated series of posttranslational events which include the postranslational conjugation and reversible phosphorylation of over 30 ubiquitin-like molecules, termed autophagy-related proteins (ATG), which trigger a series of downstream molecular events including the formation of nascent isolation membranes (phagophores), engulfment of damaged organelles by early autophagic vacuoles (AVs), the maturation of AVs, and fusion of late AVs with lysosomes to form the autolysosome, reviewed in [1,2].

**Figure 2:**
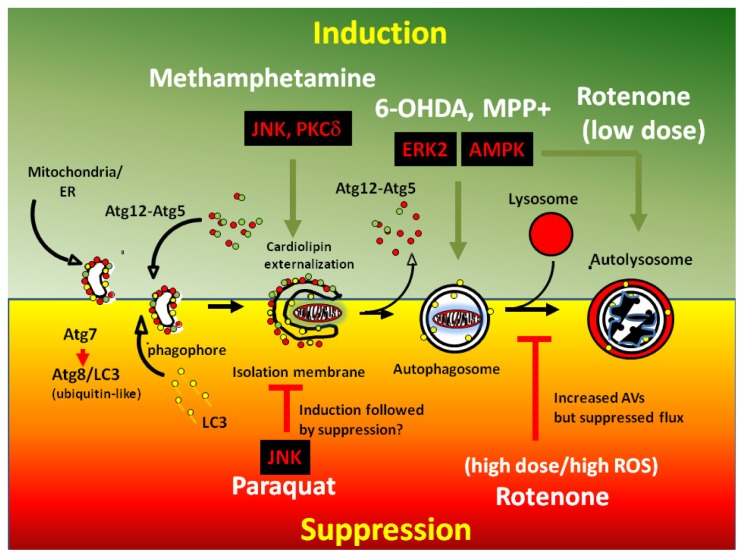
Compiled figure on the mechanisms by which PD toxins dysregulate autophagy. During physiological conditions, autophagy is a steady-state process involving both a proper level of autophagic flux and biosynthesis of organelles. Autophagy is initiated by the formation of AVs through the extension of phagophores derived from the mitochondria and endoplasmic reticulum (ER). In conjunction with ATG8(LC3), the ATG5-ATG12 complexes bind to early AVs. Early AVs sense and engulf damaged organelles, including mitochondria, for lysosomal-mediated degradation by acid hydrolases. The figure describes the ability of toxins to overactivate downstream kinase signaling pathways for dysregulating autophagy (induction shown on the upper half of the figure and suppression as shown on the bottom half of the figure) and/or lysosomal functional integrity. Please note that some toxins can elicit bidirectional responses on autophagy. For instance, activation of MAP kinases, PKCδ, and AMP kinase pathways by 6-OHDA, methamphetamine, MPP+ and low doses of rotenone can upregulate autophagic flux and lysosome-mediated degradation (green arrows in the top half of figure shown in the green background) while paraquat and high doses of rotenone can block autophagic flux and lysosome-mediated degradation (as indicated by red arrows in the lower half of figure shown in the red background) by promoting increases in ROS levels and by impairing lysosomal integrity.

The autophagy machinery is modulated by an array of well conserved and ancient molecular players that were initially characterized and identified in yeast reviewed in [29]. Repression of the mammalian target of rapamycin (mTOR) by rapamycin or by starvation inhibits ribosomal S6 kinase (p70S6) activity and elicits autophagic flux by activating class III phosphatidylinositol-3 kinase (PI-3K) which forms large molecular complexes with Beclin-1 (ATG6), a promoter of autophagy and target of ATG1, and with UVRAG protein binding partners. Following the activation of PI-3K complexes, ATG13 form complexes with ATG1 (an UNC-51-like kinase 1/2) which subsequently phosphorylates and liberates Beclin-1from Bcl-2, a negative regulator of autophagy. ATG13-ATG1 complexes then become tethered to nascent AV membranes during the initial stages of autophagy. Next, ATG5 associates with ATG12 and the resulting ATG5-ATG12 complexes become anchored to early nascent AV membranes. ATG8 (or the mammalian homolog microtubule-associated protein 1 light chain 3 (LC3)), is proteolytically processed to a lower molecular weight form by ATG4 and lipidated by ATG7 by catalytically conjugating a phosphatidylethanolamine on a *C*-terminal glycine of ATG8/LC3. These crucial posttranslational modifications allow ATG8/LC3 to bind to early AVs via its *C*-terminal lipidated glycine residue. Hence, the formation of early AVs is complete upon the timely association of ATG8/LC3 to the outer membranes of AVs. Upon maturation of the AV (ultrastructurally defined by the loss of a single membrane and AV closure), ATG5-ATG12 complexes fall off. However, ATG8/LC3 remains bound to AVs which makes this protein an ideal marker for tracking early AVs as a GFP tagged fusion protein (GFP-LC3) while the red fluorescent protein (RFP) version of LC3 (RFP-LC3) is widely employed by cell biologists as a marker for autolysosomes given that the fluorescence of the RFP moiety is highly stable inside the acidic environment of the lysosome reviewed in [30]. Finally, mature AVs fuse with lysosomes to form autolysosomes where acid hydrolases degrade biomolecules and allow for the efficient recycling of organelle components to stimulate the biogenesis of new organelles (Figure 2) [29].

ATGs are highly regulated by posttranslational modifications including protein phosphorylation. For instance, LC3 is phosphorylated by PKA at an *N*-terminal serine residue (S12) which leads to suppression of AV biogenesis in primary cortical neurons and in neuronal cell lines under physiological conditions. Oxidative stress induced by PD toxins decrease the phosphorylation of LC3. Treating neuronal cells with a chronic dose of MPP+ elevates macroautophagy by decreasing the PKA-mediated phosphorylation levels of LC3. These observations suggest that reversible phosphorylation of cytosolic LC3 by serine/threonine (ser/thr) kinases and phosphatases is a posttranslational mechanism by which PD toxins dysregulate autophagy in neurons. Other ATG proteins that are modified by proteolysis and lipidation include ATG1 and Beclin-1 while ATG13 is regulated by ser/thr phosphorylation [31,32].

To this date, only three types of mitophagy have been well described in yeast and in mammalian cells. In yeast, the outer mitochondrial membrane (OMM)-localized ATG32 interacts with the ATG11/ATG8 complex via a WXXL domain, leading to the recognition and engulfment of dysfunctional mitochondria by AVs. In red blood cells, mitophagy plays a crucial role for the elimination of mitochondria. The OMM-localized protein NIX associates with LC3 to initiate mitophagy and allows for the maturation of red blood cells by eliminating mitochondria in order to prevent the consumption of hemoglobin-bound oxygen. Lastly, a third type of mitophagy involves the interaction of two PD associated genes: the cytoprotective mitochondrially localized ser/thr kinase PINK1 and the E3 ubiquitin ligase Parkin (PARK2), reviewed in [33]. Treating neuronal cells with high doses of strong mitochondrial depolarizing agents such as carbonyl cyanide 4-(trifluoromethoxy) phenylhydrazone (CCCP) or valinomycin lead to a rapid collapse of the mitochondrial membrane potential followed by a rapid accumulation of PINK1 from the inner mitochondrial membrane (IMM) to the outer mitochondrial membrane (OMM). The synchronized loss of mitochondrial transmembrane potential in neurons induces a translocation of Parkin (a PINK1 substrate) from the cytosol to the mitochondria. At the OMM, PINK1 activates the ubiquitin ligase activity of Parkin by phosphorylation. Active Parkin then tags mitofusin 2 and VDAC for ubiquitin-proteasome-mediated degradation. Localized degradation of these proteins elicits the rapid mitochondrial translocation of LC3 and P62, a protein adaptor that links both ubiquitin-proteasome and mitophagy pathways, which ultimately leads to lysosomal-mediated degradation of mitochondria [4,34–36].

**Figure 3:**
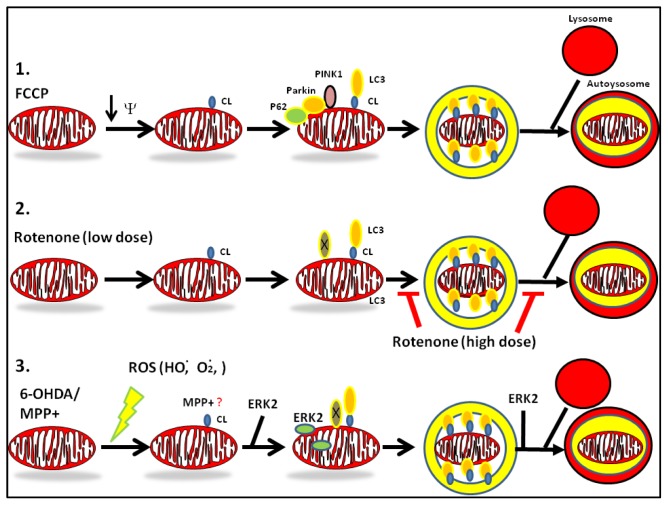
Compiled schematic figure on the effects of PD toxins on mitophagy. PD toxins 6-OHDA, MPP+, and rotenone can upregulate mitophagy. For comparative purposes, we illustrated the CCCP-mediated pathway of mitophagy (shown in top) to draw distinctions to mitophagy induced by PD-toxins. In brief, while CCCP-mediated mitophagy requires the recruitment of P62, Parkin, and PINK1 to the outer mitochondrial membrane (OMM), PD toxins elicit mitophagy through distinct mechanisms. Low doses of rotenone, 6-OHDA, and CCCP can increase autophagic flux (middle mitophagy pathway) by inducing the translocation of cardiolipin (CL) to the OMM, a process regulated by human phospholipid scramblase-3. The negative charges of CL interact with basic residues located in the *N*-terminal domain of LC3 and with other unidentified autophagic machinery components (indicated by X). On the other hand, MPP+ and 6-hydroxydopamine (lower mitophagy pathway) increase mitochondrial turnover in an ERK2-dependent manner. 6-OHDA and rotenone require externalized cardiolipin as a common signal that triggers the elimination of mitochondria in neurons [39].

The physiological role of the CCCP/Parkin-mediated mitophagy pathway in neurons *in vivo* has been the subject of high controversy. The majority of studies that characterized CCCP/Parkin-mediated mitophagy were done in non-neuronal cells including HeLa cells that transiently or stably overexpress PINK1 and Parkin. Furthermore, there are a few studies that have shown a limited involvement of Parkin and PINK1 in regulating neuronal mitophagy suggesting that divergent pathways likely exist between postmitotic and proliferating cells for triggering mitophagy [37–39]. Moreover, aside from CCCP-induced toxicity, there is no clear evidence that Parkin is involved in modulating mitophagy induced by PD toxins (Figure 3).

On the other hand, unlike its involvement as an upregulator of mitophagy induced by CCCP, transient or stable overexpression of PINK1 downregulates PD toxin-induced mitophagy in neuronal cells. Indeed, transient or stable overexpression of PINK1 blocks 6-OHDA-mediated autophagy/mitophagy and slows down the loss of mitochondria in neuronal cell lines, possibly by blocking Drp1-mediated mitochondrial fission [40]. The discrepancy on the role of PINK1 in regulating mitophagy may be a consequence of the extent of posttranslational processing and subcellular distribution of the cytosolic and mitochondrial pools of PINK1. Mitochondrial depolarizing agents block mitochondrial import and impair proteolytic processing of PINK1 which lead to a loss of lower molecular weight species of PINK1 and a rapid accumulation of OMM-localized PINK1 which phosphorylates Parkin to trigger mitophagy of irreversibly damaged mitochondria. On the other hand, partial depolarization of mitochondria by PD toxins does not block mitochondrial import of PINK1 *in vitro*. Hence, a mitochondrial pool of a lower molecular weight of PINK1 stabilizes mitochondria and blocks PD toxin-induced mitochondrial fission [41,42].

Although autophagy has been well characterized in models of starvation, our knowledge on how PD toxins dysregulate autophagy and mitophagy is still limited. In addition, many studies report contrasting observations on how PD toxins affect autophagy, suggesting that either cell and culture conditions-specific differences and bidirectional effects of toxins on autophagy or occasionally fragmentary analysis of autophagic flux can lead to wrong conclusions. While 6-OHDA and MPP+ are generally considered autophagy inducers, some studies suggest that MPP+ blocks lysosomal function at later stages of autophagic process *in vivo* [43]. Similarly, while a myriad of studies support a negative role of rotenone and paraquat on autophagy [29] (Figure 3), there are reports that demonstrate or suggest the opposite [44–46]. In the case of rotenone, the variable effects of the toxin on autophagic flux may depend on the dose, duration, and cell type analyzed *in vitro* and *in vivo*. This phenomenon can be partly explained by the level of ROS and extent of mitochondrial damage induced by a PD toxin [17,18,39,47–49].

How do neurons sense damaged mitochondria for removal by mitophagy? The specific signals that trigger the cargo recognition of damaged mitochondria by the autophagy machinery have eluded cell biologists for several decades. Loss of the mitochondrial transmembrane potential and activation of the permeability transition pore (PTP) were initially postulated as cellular signals that trigger mitophagy in injured liver cells [50,51]. However, these factors alone do not act as elimination signals for mitochondria since PD toxins can promote mitophagy in the absence of significant loss of transmembrane potential or import. Recently, one particular *in vitro* study characterized the “mitophagic” potential of several PD toxins and unveiled a novel molecular mechanism that explains how the autophagy machinery senses dysfunctional mitochondria in neurons. Indeed, by employing a series of elegant mass spectrometry and single cell image analysis experiments, Chu *et al*., 2013 demonstrated that treating neurons with PD toxins induce a rapid translocation of the anionic phospholipid cardiolipin (CL) from the inner mitochondrial membrane (IMM) to the OMM. Externalized CL then recruits LC3 at the OMM by interacting with its predominantly positively charged N-terminal region to initiate mitophagy. Unexpectedly, both Parkin and PINK1 are dispensable for mitophagy induced by PD toxins (6-OHDA and rotenone) suggesting that other unidentified downstream molecular players and non-canonical pathways govern PD toxin-mediated mitophagy (Figure 3) [52].

## 3. Convergence of PD Toxins and Genetic Models of PD: Role of ROS

There are several lines of evidence that suggest that PD toxins and genetic models of PD converge on mitochondrial dysfunction and dysregulated autophagy/mitophagy. For instance, RNAi-mediated loss of endogenous PINK1 and DJ-1 elicit robust dynamin related protein 1 (Drp-1)-mediated mitochondrial fission, increased mitochondrial ROS, mitochondrial dysfunction, and dysregulated autophagy in neurons suggesting that both PD genes are critical for maintaining mitochondrial and autophagic homeostasis. While only a few *in vitro* studies suggest that PINK1 is a negative regulator of autophagy and mitophagy, there are more studies that implicate PINK1 as an upregulator of mitophagy that cooperates with Parkin to remove CCCP-damaged mitochondria. Hence, the apparent contradictory roles of PINK1 on autophagy/mitophagy may depend on the extent of mitochondrial depolarization, type of toxin used, posttranslational processing of PINK1, and cell type analyzed during the study [9,21,36]. Furthermore, gain of function mutations in LRRK2, a large WD repeat ser/thr kinase associated with autosomal dominant forms of PD, increases ERK1/2 signaling, elevates macroautophagy, promotes an imbalance of calcium handling by mitochondria, and promotes mitophagy of dendritic mitochondria, a pathological event that precedes the retraction of neurites in primary cortical neurons. These results suggest that LRRK2 is an upregulator of autophagic flux in neurons [12,53]. Conversely, loss of DJ-1 function impairs basal autophagy, a pathological event that is associated with an aberrant accumulation of damaged mitochondria that leads to the excess release of mitochondrial superoxide to the cytosol and elicits mitochondrial fission [54]. Finally, RNAi-mediated knockdown of endogenous ATP13A2 *in vitro* (a lysosomal P-type ATPase associated with familial PD) or expression of PD-associated mutations of ATP13A2 promote modest mitochondrial fragmentation, lead to impaired mitochondrial flux, and cause an aberrant accumulation of dysfunctional mitochondria, and concomitant impaired biogenesis [55,56]. Hence, in conjunction with mitochondrial dysfunction, both PD toxins and genetic models of PD can either impair or overactivate autophagic flux in neurons.

The aforementioned observations pose the following elusive question: Why do some PD toxins and genetic models of PD elicit bidirectional responses on autophagic flux? The answer may lie in how ROS dysregulates certain kinase signaling pathways that govern autophagy/mitophagy. Hence, the type and concentration of ROS (superoxide: O_2_^−^, hydrogen peroxide: H_2_O_2_, hydroxyl: ·HO) or reactive nitrogen species (RNS) (nitrogen dioxide: NOO·) can tilt the balance to either activation or suppression of autophagy. In general, high levels of ROS impair autophagic flux while a transient increase in ROS levels triggers autophagy by activating/inactivating redox-sensitive pathways. For instance, high ROS levels can impair autophagy by preventing the activation of the UVRAG/Beclin-1 complex, leading to decreased AV biogenesis, fusion of AVs with lysosomes, and by promoting lysosomal dysfunction [43]. On the other hand, low levels of ROS can enhance autophagy by activating specific ser/thr kinase signaling pathways such as extracellular regulated kinase 1/2 (ERK1/2), c-JUN *N*-terminal kinase-1(JNK1), and AMP-activated protein kinase (AMPK) pathways and by concomitantly inactivating redox-sensitive phosphatases, reviewed in [57,58]. Moreover, treating neuronal cells with 6-OHDA transiently increases mitochondrial and cytosolic ROS levels, leads to enhanced phosphorylation of ERK1/2 and mitochondrial translocation of p-ERK1/2 to drive mitophagy [6,59]. Moreover, elevated ROS levels induced by MPP+ upregulate autophagy by activating CDK5 to upregulate autophagic flux while *S*-nitrosylation of Parkin inhibits its ubiquitin-ligase activity and impairs its ability to promote CCCP-mediated mitophagy [57]. Regardless of the final outcome on the autophagic status of neurons, a significant loss of mitochondrial levels as a result of prolonged dysregulated autophagic flux or excessive ROS levels (presumably due to an accumulation of dysfunctional mitochondria) is equally detrimental to neuronal survival.

The ensuing sections of this review will not only highlight the various pathological effects of five well characterized PD toxins (MPP+, rotenone, 6-OHDA, paraquat, and methamphetamine) on mitochondrial function and autophagy/mitophagy, but we propose several mechanisms by which PD toxins dysregulate autophagy.

## 4. PD Toxins and Their Mechanisms of Autophagy

### 4.1. MPTP

MPP+ is a cationic metabolite of the neurotoxin1-methyl-4-phenyl-1,2,3,6-tetrahydropyridine (MPTP) [60]. In 1982, MPTP was originally identified as a contaminant of MPPP, an analog of meperidine, that was accidently ingested by several Californian young adults who rapidly experienced Parkinsonian-like symptoms including tremors and loss of muscle coordination [60]. Unfortunately, the neurologist who reviewed these cases concluded that the loss of motor function was due to the rapid and selective destruction of midbrain neurons caused by MPTP intoxication [61]. Today, MPTP is used as a *bona fide in vivo* chemical model of PD. Injecting mice with acute doses of MPTP rapidly induces the degeneration of *substantia nigra* neurons leading to the onset of PD symptoms including a loss of gait, muscle rigidity, and motor dysfunction. Moreover, MPTP intoxication faithfully reproduces PD pathology in rodents including a modest accumulation of protein aggregates and mitochondrial dysfunction [62]. The pathological effects of MPTP toxicity can be partly ameliorated by cotreating mice with monoamine oxidase B (MAO-B) inhibitors suggesting that secondary metabolites are the causative agents for the degeneration of midbrain dopamine neurons [63,64]. While MPTP does not directly cause midbrain degeneration *in vivo*, the toxin efficiently crosses the blood brain barrier where it is metabolically transformed to MPP+, the destructive form of the toxin, by glia. Moreover, MPP+ is actively transported inside dopaminergic neurons via the dopamine active transporter (DAT) since treating cells with pharmacological inhibitors of DAT protects cells from MPP+-induced cell death [63–66]. Mechanistically, MPP+ blocks oxidative phosphorylation at the level of complex I and increases the steady state levels of mitochondrial superoxide. Remarkably, complex I inhibition by MPP+ is not required for promoting its neurotoxic effects since neurons that are deficient for functional complex I show similar toxicity to toxin-treated wild-type neurons suggesting that alternative mechanisms mediate the neurotoxic effects of MPP+ [67].

Other pathological effects of MPP+ that have been described in several cell culture studies of PD include synaptic dysfunction, α-synuclein aggregation, impairment of retrograde/anterograde transport, mitochondrial swelling and loss of cristae, upregulation of autophagy, lysosomal expansion (increased lysosomes per neuron), lysosomal dysfunction, and caspase 3-independent cell death [17,43,68–70] (Figure 1). The early induction of AVs by MPTP *in vivo* or its active metabolite MPP+ *in vitro* has been shown to be mediated by cdk5-dependent phosphorylation of endophilin, and by regulating the vps34-Beclin-1 complex [16]. However, unlike 6-OHDA, MPP+-mediated neurotoxicity does not elicit Drp1-dependent mitochondrial fragmentation but promotes robust mitochondrial swelling by activating the permeability transition pore complex since treating cells with cyclosporine restores normal mitochondrial morphology in toxin co-treated cells [71].

#### Mechanisms of Dysregulation of Autophagy by MPP+

MPP+ can increase autophagic flux in SH-SY5Y cells, a dopaminergic cell line widely used as a cell culture model of PD that expresses tyrosine hydroxylase (TH) and DAT receptors upon differentiation with retinoic acid [6,17,18]. Treating SH-SY5Y cells with MPP+ transiently increases ERK signaling and upregulates autophagy and mitophagy in neurons. MPP+-mediated increases in MAP kinase signaling are required for eliciting autophagic flux since treating cells with pharmacological inhibitors of MEK (U0126) during the early phases of MPP+-induced autophagy induction blocks autophagy and mitophagy and increases cell survival. Interestingly, treating cells with MEK inhibitors after 12 h of toxin treatment did not promote protection, suggesting that autophagic suppression is likely involved at later time points of toxin treatment *in vitro* [17] (Figures 2 and 3). These observations suggest that MPP+-mediated ERK activation promotes mitochondrial dysfunction and impairs mitochondrial biogenesis [18]. Mechanistically, MPP+-mediated upregulation of autophagy operates through a non-canonical pathway that is distinct from starvation-induced autophagy as siRNA-mediated knockdown of Beclin-1 or pharmacological inactivation of class III PI-3 kinases by wortmannin or 3-methyladenine (3-MA) do not block toxin-induced autophagy and mitophagy [17]. Hence, future studies are required to identify the downstream molecular players that regulate MPP+-mediated autophagy and mitophagy in neurons.

Conversely, several studies have recently challenged the view that MPP+ is a positive regulator of autophagy. Two cell culture studies showed that MPP+ blocks autophagy as evidenced by increased steady-state levels of P62, increased AV numbers along with lysosomal depletion and dysfunction presumably due to leakage of lysosomes, impaired lysosomal biogenesis, and increased proteasomal-mediated degradation of proteins [43,72,73]. However, these studies did not examine the temporal dynamics of autophagic flux and mitophagy. Hence, it is plausible that prolonged MPP+ toxicity *in vitro* can lead to a transient increase in autophagic flux followed by an irreversible suppression on autophagy due to high levels of ROS. In further agreement with this concept, rapamycin treatment and overexpression of TFEB, a nuclear transcription factor, increases lysosomal biogenesis and cell survival and these protective effects are more efficient during the late time points of MPTP or MPP+ treatment *in vitro* and *in vivo* [43]. Alternatively, the inconsistent effects of MPP+ on autophagy may be attributed to differences observed between immortalized cell lines and primary neurons. However, it is important to recognize that *in vivo* data support a negative role of MPTP on autophagic flux. Indeed, *in vivo*, suppression of autophagic flux induced by MPTP is detrimental to neuronal survival as treating mice with the autophagy inducer rapamycin after seven days of MPTP treatment significantly increases the number of surviving dopamine neurons and decreases the levels of α-synuclein aggregates [74]. Hence, it is likely that the protective effects of rapamycin are more effective when autophagic suppression occurs during late time points of toxin treatment.

LC3 is phosphorylated by PKA and PKC, leading to suppression of macroautophagy [75,76]. A mechanism by which MPP+ dysregulates macroautophagy in neurons involves decreasing the PKA-mediated phosphorylation levels of LC3. Indeed, treating primary cortical neurons or SH-SY5Y cells with a bolus of MPP+ decreases the phosphorylation of LC3 and promotes autophagy-mediated neurodegeneration. In further agreement with this observation, transient expression of PKA phosphomimetic mutants of LC3 protect SH-SY5Y cells against MPP+-induced autophagic stress by blocking macroautophagy while transient expression of PKA phospho-deficient LC3 mutants produce the opposite effect [75]. Alternatively, the detrimental effects of MPP+ can be partially reversed by co-treating cells with pharmacological activators of PKA (dibutyryl cyclic-AMP and forskolin). These observations suggest that elevated autophagic flux by MPP+ involves dephosphorylation of LC3 by an unidentified ser/thr phosphatase [75].

### 4.2. Rotenone

Rotenone is an odorless and lipophilic naturally occurring pesticide and is a widely popular chemical model of PD [77,78]. Rotenone was first isolated in 1865 from *Robinia nicou* by a French botanist. Today, this natural compound is industrially used as a pesticide and as a fish poison [79]. Rotenone is classified as a *bone fide* inhibitor of complex I since treating neurons with this toxin impairs basal and maximal oxygen consumption, decreases the mitochondrial transmembrane potential, and depletes mitochondrial-ATP levels. Like MPP+, the notion that complex I inhibition is required for mediating the neurotoxic effects of PD toxins has been recently challenged suggesting that the neurotoxic effects of rotenone likely involves an increase in ROS levels *in vitro* [67]. *In vivo*, chronic injections of rotenone in Lewis rats faithfully reproduces many neurochemical and behavioral alterations of PD including loss of *substantia nigra* neurons, robust extra- and intra-neuronal accumulation of protein aggregates, mitochondrial swelling and dysfunction, decreased release of dopaminergic vesicles, and time-dependent loss of motor function and coordination [77,80–82]. Moreover, complex I inhibition induced by rotenone leads to activation of the permeability transition pore, mitochondrial swelling, decreases mitochondrially-derived ATP levels, and induces caspase 3-independent cell death *in vitro* [81]. Interestingly, the *in vivo* neurotoxic effects of rotenone have been associated with the production of superoxide released by mesencephalic midbrain microglia which damage neighboring midbrain neurons. These observations suggest that a microglia-neuron interaction is a mechanism by which rotenone selectively targets dopaminergic neurons [83]. Beyond mitochondrial damage, rotenone suppresses the activation of various prosurvival gene transcription programs, decreases the levels of cytoprotective proteins involved in antioxidant response and organelle quality control pathways (Parkin and DJ-1), decreases Parkin levels, and produces metabolic catastrophe in neurons due to a rapid loss of mitochondrial ATP levels [47,82,84].

#### Mechanisms of Dysregulation of Autophagy by Rotenone

Rotenone can produce bidirectional effects on autophagic flux with similar detrimental effects to neuronal survival. Rotenone blocks autophagy in most instances since treating mice with kaempferol or with rapamycin—strong inducers of autophagy—robustly protects against rotenone-mediated toxicity [47,84]. In further support of this view, treating SH-SY5Y cells with high doses of rotenone blocks ATG5-dependent autophagy which leads to lysosomal dysfunction, increased P62 levels, and an aberrant accumulation of α-synuclein [47,48]. On the other hand, only a few studies have implicated rotenone as an inducer of autophagic flux (Figure 2). For instance, treating HeLa cells with rotenone induce cell death by upregulating autophagy and mitophagy, a process that is triggered by mitochondrial superoxide [49]. Moreover, increased autophagic flux has been observed in primary cortical neurons treated with low doses of rotenone as evidenced by a decrease in the steady-state levels of P62 and rapid loss of mitochondria that can be reversed by co-treating cells with bafilomycin A2, a specific inhibitor of vacuolar-type H(+)-ATPase, or by RNAi-mediated knockdown of ATG7 and ATG8/LC3. The mechanism by which LC3 recognizes damaged mitochondria in rotenone-treated neurons involves the externalization of cardiolipin to the OMM by human phospholipid scramblase-3. Externalized cardiolipin then recruits LC3 at the mitochondria and other unidentified downstream proteins to initiate mitophagy and lysosomal-mediated degradation of mitochondria. Interestingly, rotenone-induced mitophagy does not involve the participation of P62, PINK1, or Parkin (Figure 3) [39]. Hence, the discrepancies on the effects of rotenone on macroautophagy may be attributed to differences in the dosage, the duration, and cell type which can produce variable levels of ROS and mitochondrial damage [39,47–49,84,85].

Although the molecular mechanisms by which rotenone can block macroautophagy remain to be elucidated, it is conceivable that rotenone-mediated suppression of autophagic flux may be a consequence of the following factors: a rapid loss of mitochondrial ATP levels which is necessary to maintain the proper function of lysosomes by powering ATP-dependent acid hydrogen pumps, and redox-inactivation of the protease activity of ATG4 which leads to impaired maturation of ATG8/LC3.

### 4.3. 6-Hydroxydopamine (6-OHDA)

6-OHDA is modest inhibitor of complex I that promotes the selective degeneration of midbrain neurons by eliciting increases in mitochondrial and cytosolic ROS [86]. 6-OHDA is a redox cycling chemical analog of dopamine that selectively destroys *substantia nigra* neurons with a single bolus or by chronic treatment *in vivo*. Interestingly, unlike other PD toxins, 6-OHDA’s toxicity does not predominantly involve mitochondrial dysfunction or loss of steady state levels of NAD+ but likely involves an increase in ROS levels which cause extensive oxidative damage in neurons beyond the mitochondrion. Upon import by DAT receptors in dopamine neurons [87], 6-OHDA rapidly undergoes rapid auto-oxidation (~15 min), and produces a lethal “soup” of ROS species including hydroxyl radicals, hydrogen peroxides, peroxynitrites, and a transient increase in mitochondrial superoxide in neurons [88,89]. Moreover, 6-OHDA elicits Drp1-dependent mitochondrial fission, loss of mitochondrial content by upregulating Beclin-1 independent mitophagy, and alters microtubule dynamics [59,90,91]. Unlike other PD toxins, 6-OHDA causes global repression of prosurvival transcription programs including decreased cyclic AMP-regulated protein kinase signaling, promotes the mislocalization of prosurvival transcription factors from the nucleus to the cytosol [92], and blocks nuclear import of prosurvival transcription factors in SH-SY5Y cells [93–95].

#### Mechanisms of Dysregulation of Autophagy by 6-OHDA

The effects of 6-OHDA on macroautophagy and mitophagy have been very well characterized in SH-SY5Y cells, primary neurons and *in vivo*. Treating neurons with 6-OHDA elicits macroautophagy as quantified by image analyses of cells transfected with GFP-LC3, a fluorescent reporter of autophagy, and as assessed by quantifying the amount of immunoreactive lipidated LC3 (LC3-II). Interestingly, some of the cells intoxicated by 6-OHDA show the presence of abnormally large ring-like structures of LC3 suggesting that 6-OHDA toxicity not only leads to increased AV numbers per cell but increased AV size as well, a morphological hallmark of enhanced autophagic flux [6]. In mice, intrastriatal injection of 6-OHDA elicits macroautophagy and this pathological event can be suppressed by adenoviral-mediated transduction of Akt suggesting that Akt is a suppressor of toxin-mediated autophagy *in vivo* [96]. Moreover, treating neuronal cells with a single LD50 dose of 6-OHDA increases p-ERK1/2 levels which precede Drp1-mediated mitochondrial fission, elevates macroautophagy with ensuing loss of mitochondrial levels, promotes lysosomal expansion, and elicits caspase 3-dependent apoptosis. Mechanistically, increased levels of H_2_O_2_ elicited by 6-OHDA increases autophagic flux since treating cells with *N*-acetyl cysteine not only suppresses the increased ROS levels but blocks cell death and autophagy induced by 6-OHDA *in vitro* [97]. Secondly, the translocation of p-ERK1/2 to mitochondria is sufficient to promote 6-OHDA-mediated mitophagy since transient overexpression of constitutively active constructs of ERK2 or of MEK in SH-SY5Y cells are sufficient to recapitulate toxin-induced mitophagy and elevated basal cell death (Figure 3) [6]. On the other hand, 6-OHDA-mediated autophagic flux involves ROS-mediated activation of ERK1/2 and AMP kinase signaling pathways (Figure 2) [17,97]. Like MPP+, 6-OHDA-mediated upregulation of autophagic flux plays a detrimental role since treating SH-SY5Y cells with bafilomycin A1, mitochondrially directed antioxidants, or with ATG7 and ATG8siRNA partially blocks cell death and loss of mitochondrial proteins induced by 6-OHDA [6,98].

### 4.4. Paraquat

In conjunction with rotenone, paraquat (*N*,*N*′-dimethyl-4,4′-bipyridinium dichloride) is one of the two most widely used non-selective plant pesticides to date. Like rotenone, a few epidemiological studies have linked prolonged exposure of paraquat to increased risk for developing PD in humans [99]. Mechanistically, paraquat inhibits mitochondrial oxidative phosphorylation and blocks the release of dopamine from synaptic terminals prior to neurodegeneration [100,101]. However, unlike other PD toxins, paraquat toxicity robustly promotes protein aggregate formation and genetically interacts with α-synuclein to exacerbate PD pathology *in vivo* [102,103]. Paraquat-induced increases in α-synuclein aggregate levels is likely due to impairment of autophagic flux since treating mice with paraquat alters the LC3 II to LC3 I ratio in animal brains [19]. In intoxicated dopamine neurons, paraquat blocks autophagy as evidenced by an increase in the number of AVs per cell due to impaired basal ATG5-dependent autophagy and promotes an increased accumulation of α-synuclein [104]. Mechanistically, paraquat-mediated autophagy is initiated by elevating JNK1 signaling but eventually comes to halt due to increased ROS levels. Mechanistically, an increase in mitochondrial ROS produced by oxidoreductase-2 may underlie the molecular etiology of paraquat-induced neurotoxicity in midbrain neurons as treating neurons with a pharmacological oxidoreductase inhibitor or siRNA against oxidoreductase-2 potently protects against paraquat-toxicity *in vivo*. These results and our unpublished observations suggest that mitochondrial ROS elicited by paraquat blocks autophagy both *in vitro* and *in vivo* [105]. In further support of this concept, treating neurons with rapamycin and other inducers of autophagy robustly confer neuroprotection against paraquat-induced toxicity *in vitro* and *in vivo* [19,84,106]. However, some other studies suggest the opposite effect of paraquat on autophagy. The analysis of AVs and LC3 levels in SH-SY5Y cells in response to paraquat suggests a pro-autophagic effect induced by the toxin [107].

Future studies are required to identify the molecular players downstream of JNK1 that regulate paraquat-mediated autophagic flux. However, it is likely that JNK1 signaling initially elevates autophagy, but it eventually comes to halt due to increased ROS levels or other yet unidentified negative signals (Figure 2). Other proposed mechanisms by which paraquat promotes neurotoxicity involve a progressive loss of soluble Parkin, increased Parkin aggregation, and loss of its cytoprotective activity *in vitro* and *in vivo* [108]. Moreover, it is not clear how paraquat promotes mitochondrial dysfunction and whether paraquat suppresses protective Parkin-mediated mitophagy in dopamine neurons. All these questions are critical for not only advancing our understanding as to how paraquat dysregulates autophagy in PD but will help advance the development of new pharmacological activators of autophagy as therapeutic alternatives for treating PD in humans affected by chronic paraquat exposure.

### 4.5. Methamphetamine

Methamphetamine is a psychoactive drug that has been associated with increased risk of PD [109]. Methamphetamine inhibits the reuptake of dopamine at the synapse by inhibiting the DAT receptor [110]. Moreover, protracted abuse of this drug leads to increased cytoplasmic levels of dopamine due to a rapid impairment of the vesicular monoamine transporter leading to increased ROS levels, mitochondrial dysfunction, and a selective decrease of antioxidant defenses [111,112]. Mysteriously, this myriad of pathological factors induced by methamphetamine leads to the selective destruction of neurites [113,114] while sparing cell bodies of neurons suggesting that neurites are the primary neurotoxic target of methamphetamine *in vitro* (Figure 1) [115].

Unlike 6-OHDA and MPP+, increased autophagic flux induced by methamphetamine plays a protective role for the timely removal of protein aggregates and dysfunctional mitochondria [116–119]. In further support of this view, methamphetamine toxicity is partially reversed by treating cells with the pharmacological autophagy activator rapamycin while treating neurons with bafilomycin A1 exacerbates toxicity *in vitro* [117]. Mechanistically, methamphetamine elicits autophagic flux by activating JNK1 which phosphorylates Beclin-1 and promotes its dissociation from Bcl-2 leading to over-activation of class III PI-3 kinase-dependent autophagy in midbrain neurons [120]. While Parkin is a target of methamphetamine, one study suggested that overexpression of Parkin *in vivo* is cytoprotective as adenoviral-mediated overexpression of Parkin in striatal and *substantia nigra* confers resistance of dopamine neurons to methamphetamine-mediated toxicity. Hence, increasing Parkin levels and/or activity could be a disease-modifying strategy for treating sporadic PD [121,122]. Another ser/thr kinase that modulates methamphetamine-induced autophagy is protein kinase *C*-δ. Methamphetamine toxicity elicits phosphorylation and proteolytic processing of PKC-δ which translocates to mitochondria to promote mitophagy and macroautophagy *in vitro* (Figure 2) [123]. Although our current understanding of methamphetamine-mediated neurodegeneration has increased over the past decade, future experiments are required to determine whether methamphetamine promotes mitophagy at the synaptic terminals, how compensatory protective macroautophagy delays neuronal cell death, and to identify downstream effectors that regulate methamphetamine-induced mitophagy.

## 5. Environmental Exposure and Prevention

Among environmental exposures, exposure to pesticides and certain metals have been consistently linked to increased risk of PD.

As described in the previous section, pesticides such as rotenone and paraquat have repeatedly resulted in PD-like pathology in animal models. However, only a handful of epidemiological studies have suggested that humans chronically exposed to pesticides show a significantly increased risk for developing idiopathic forms of PD and Lewy body dementias. For instance, an epidemiological study done on field workers who were exposed to pesticides and lacked proper protective gear while at work showed a 70% higher chance of developing PD compared to age-matched control groups [124]. Another study solved the problem of identifying pesticide exposures to specific individuals through the use of California Pesticide Use Reports. This study in California’s Central Valley found that, among the general population, exposure to the pesticides maneb and paraquat increased Parkinson’s disease risk, particularly among those exposed at a young age [125]. Another study found that a combined exposure to ziram and paraquat was associated with an 80 percent increase in the risk of developing PD. Furthermore, other studies have shown that increased exposure to these two pesticides plus maneb increased the risk of developing PD by 300 percent [126]. Furthermore, researchers at the Parkinson’s Institute and Clinical Center in California reported that pesticide applicators that used paraquat or rotenone showed a two-fold risk of developing Parkinson’s disease [127]. When researchers compared the lifetime histories of the patients and the control subjects, they found that people who had handled or applied pesticides in the home or garden were 70 percent more likely to develop PD than those who had not received such exposure. Occupational exposure to specific metals (manganese, copper, lead, iron, mercury, zinc, aluminum and others) appears to be a risk factor for developing PD in some, but not in all case-control studies. In a population-based case-control study by Gorell *et al.*, the authors found that subjects with more than 20 years of exposure showed a significantly increased association between copper (OR = 2.49, 95% CI = 1.06, 5.89) and manganese exposure with PD (OR = 10.61, 95% CI = 1.06, 105.83). These findings suggest that chronic exposure to these metals is associated with PD, and that they may act alone or synergistically over time to help produce PD [128]. Another study by Coon *et al*. investigated the association between objective chronic occupational lead exposure and the risk of PD. The study found that risk of PD was elevated by more than two-fold for individuals in the highest quartile for lifetime lead exposure relative to the lowest quartile, after adjusting for age, sex, race, smoking history, and coffee and alcohol consumption [129]. However, long-term exposure to metals is not easily measured, and the results of studies measuring PD risk and specific metals have been inconsistent. For instance, a population-based, case-control study of 404 incident PD cases and 526 age and sex-matched controls, showed that collecting self-reported work histories including job titles and exposures to various industrial toxicants found that risk to PD was not significantly affected by farming work, by metal work, or by exposure to metals, or solvents [130]. Just like pesticides, metal-based nanoparticles also appear to play a role in the regulation of autophagy. For instance, manganese (Mn^+2^) nanoparticles have been reported to induce the loss of TH positive dopaminergic neurons and neuronal processes in primary mesencephalic cultures. Mn^+2^ nanoparticles effectively enter dopaminergic neuronal cells and exert neurotoxic effects by activating pro-apoptotic signaling and autophagy (increased Beclin-1 cleavage and expression of LC3) [131]. These observations underscore the need for assessing possible health risks associated with an increased use of nanoparticles in modern applications. Similar findings have been reported for copper oxide nanoparticles and the copper compound Casiopeina III-ia [132,133].

Hence, the overarching goal of all scientific research is two-fold: prevent disease and treat it effectively. PD is a complex multifactorial disease involving the interaction of age, environmental factors, and genetics. The first step in prevention of idiopathic PD will undoubtedly be the need to characterize and identify all potential PD toxins and subsequently reduce, if not prevent unnecessary exposure of the general population. Identification of susceptible populations, increased awareness of the risks and community-level health education, and consistent toxicological and epidemiological data will all go hand-in-hand towards developing an effective prevention strategy. The lack of knowledge and epidemiological data underscores the need to implement new public health initiatives to prevent the risk of developing PD and other neurodegenerative disorders. For instance, farm workers and other field workers who spray pesticides should be required to wear protective gear including respirators, gloves and garbs. The number of pesticides, and their concentration and combinations should be thoroughly regulated. Since exposure is directly correlated with duration, there should be a limit on the number of hours a day spent spraying pesticides. Furthermore, relocation programs supported at the local or state level should allow susceptible high risk populations to move from geographical locations harboring large chemical plants to safer areas in order to lower incidences of PD and other neurodegenerative diseases.

In terms of therapeutics, new innovative technologies that analyze mitochondrial dysfunction and induction of autophagy should be at the forefront of the health sciences field to facilitate an early and rapid diagnosis of PD prior to the onset of symptoms. Much progress has been made in the identification and development of compounds to induce or suppress autophagy. These agents fall into two main categories based on mechanism of action, that is, whether they work through an mTOR-dependent or independent pathway [134]. Some of the mTOR-dependent autophagy inducers include Rapamycin, Rottlerin, Torin1 and Dexamethasone, while Lithium, Verapamil, Clonidine, and Spermidine are a few of the mTOR-independent autophagy inducers. Although there are autophagy-modulating compounds that are already approved for use in humans, reviewed in [135], some are not optimal for clinical use. Other innovative technologies that may be used by hospitals and health clinics include performing extracellular flux assays using a Seahorse Biosciences platform or Clark electrodes in patient-derived fibroblasts or white blood cells for quantifying mitochondrial respiration and oxidative stress in response to exposure to environmental intoxicants [136,137]. At the same time, autophagic stress in patient-derived fibroblast can be assessed by quantifying the number of GFP-LC3 and RFP-LC3 puncta per cell by transfecting the tandem reporter GFP-RFP-LC3 construct to correlate mitochondrial respiration deficits with dysregulated autophagy in PD [138].

## 6. Concluding Remarks

In summary, an increased chronic exposure of humans to PD toxins along with interactions with certain genes and aging can increase the risk of developing PD. The pathophysiological spectrum of PD toxins include a convergence of mitochondrial dysfunction, increased ROS, and dysregulation of autophagic pathways in neurons but differ on their ability to promote oxidative damage beyond the mitochondrion. However, several clear mechanistic differences exist among PD toxins on their ability to dysregulate autophagy and mitophagy in neurons including the selective activation of MAP kinase and JNK-regulated signaling pathways. Increasing our understanding on the mechanisms by which environmental toxins dysregulate autophagy/mitophagy will hence, be critical for developing novel PD diagnostics and therapies. Moreover, PD therapies that can increase cytosolic PKA or PKC signaling can be developed to suppress overactive autophagy/mitophagy in order to prevent the premature rapid loss of mitochondria while allowing for biogenesis to take place. Alternatively, in 2008 the FDA approved 14 autophagy-inducing and seven autophagy-suppressing compounds that act through mTOR-independent pathways [139]. These compounds appear to act on a variety of pharmacological targets, including imidazoline receptor agonists, l-type calcium channel antagonists and calpains. Alternatively, rapamycin analogs that lack unwanted immunosuppressive effects can be developed to stimulate autophagic flux in order to remove intra-neuronal protein aggregates induced by PD. Finally, as the human population ages and is increasingly exposed to unidentified endogenous and uncharacterized artificially-generated PD mimetics, we have to be mindful of the pressing need to enhance public education, financially boost toxicology research, and implement health initiatives to mitigate the risk of developing PD in the near future.
